# Dosimetric advantages of stereotactic radiosurgery as a boost to adjuvant conventional radiotherapy in the setting of adenoid cystic carcinoma of the parotid with skull base invasion

**DOI:** 10.1002/ccr3.1788

**Published:** 2018-09-23

**Authors:** Jae L. Phan, Courtney Pollard, He Wang, Sweet Ping Ng, Tommy Sheu, Lawrence E. Ginsberg, Amy C. Hessel, Paul W. Gidley, David I. Rosenthal, Jack Phan

**Affiliations:** ^1^ Rice University Houston Texas; ^2^ Department of Radiation Oncology MD Anderson Cancer Center Houston Texas; ^3^ Department of Radiation Physics MD Anderson Cancer Center Houston Texas; ^4^ Department of Diagnostic Radiology MD Anderson Cancer Center Houston Texas; ^5^ Department of Head and Neck Surgery MD Anderson Cancer Center Houston Texas

**Keywords:** adenoid cystic carcinoma, boost, gamma knife, skull base, stereotactic radiosurgery

## Abstract

This study highlights gamma knife stereotactic radiosurgery (GK‐SRS) as boost therapy in a patient with adenoid cystic carcinoma of the parotid involving the skull base and invasion of the facial nerve. Using GK‐SRS, dose to the brainstem and temporal lobe were reduced when compared to less conformal radiotherapy techniques.

## 1 INTRODUCTION

Gamma knife stereotactic radiosurgery (GK‐SRS) as a boost theoretically offers the potential of reduced side effects compared to photon boost plans when used for salivary malignancies of the head and neck with skull base involvement that is often secondary to perineural invasion (PNI).^1^, ^2^ Here, we present a case of adenoid cystic carcinoma (ACC) of the parotid with PNI that tracked through the stylomastoid foramen superiorly to the level of the geniculate ganglion. This patient was treated with surgical resection, adjuvant up‐front GK‐SRS boost (GK‐B) to skull base disease, followed by concurrent chemoradiation to the postoperative parotid bed and skull base.

## 2 CASE

**Figure 1:**
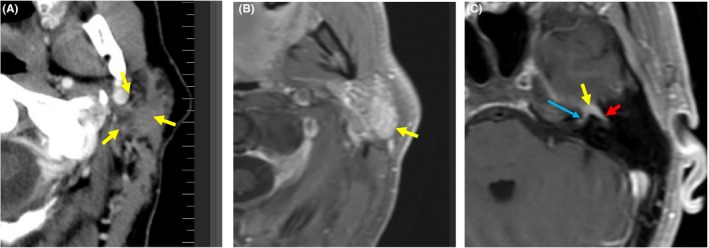
Pretreatment imaging. A, Axial computed tomography image status post contrast administration demonstrating nonenhancing mass in the left parotid gland (arrows). B, Axial T1‐weighed fat‐suppressed postgadolinium MR image demonstrates enhancing mass (arrow). C, Axial postcontrast fat‐suppressed T1‐weighted MR image demonstrating tumor involvement of the geniculate ganglion (yellow arrow), labyrinthine (blue arrow), and tympanic (red arrow) segments of the left facial nerve

A 44‐year‐old Caucasian male presented with left facial swelling and otalgia. Physical examination demonstrated a left parotid mass. Computed tomography (CT) scan and magnetic resonance imaging (MRI) demonstrated a potential malignant lesion (Figure 1A,B), with involvement of the geniculate ganglion, labyrinthine, and tympanic segments of the left facial nerve was also identified (Figure 1C). Imaging did not demonstrate any lymph nodes in the neck.

**Figure 2:**
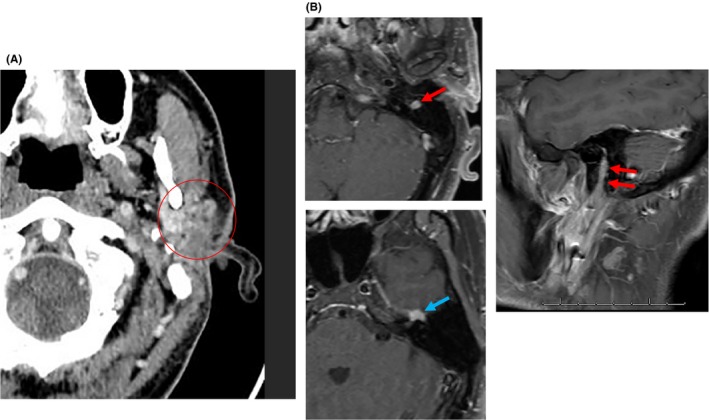
Postoperative imaging. A, Axial postcontrast CT image demonstrating nonspecific postoperative changes in the left parotid bed (red circle). B, Left (axial) and sagittal (right) postcontrast fat‐suppressed T1‐weighted MR images demonstrating enhancing perineural tumor spread along the descending or mastoid facial nerve segment (red arrows) extending to the geniculate ganglion (blue arrow)

The patient underwent a left total parotidectomy with sacrifice of the left facial nerve. Pathology revealed a 4.5 cm ACC with PNI, extension into intraparotid lymph nodes, and positive surgical margins. A postoperative CT scan demonstrated no gross residual disease in the parotid bed or neck (Figure 2A) but enhancing perineural spread of tumor was noted along the mastoid facial nerve segment extending to the geniculate ganglion (Figure 2B).

After multidisciplinary discussion, additional surgery was not favored due to treatment morbidity and a high risk of leaving behind residual disease. It was decided that an upfront GK‐B to the disease in the skull base followed by concurrent chemoradiation would reduce radiation dose to the temporal lobe and brainstem compared to a simultaneous integrated photon boost plan. The GK‐B was delivered upfront and prior to conventionally fractionated radiation because the invasive GK‐SRS head frame would be better tolerated in the absence of radiation dermatitis.

Regarding GK‐SRS treatment planning, the skull base and perineural disease including the geniculate ganglion and internal auditory canal was treated to 10 Gy prescribed to 50% isodose line utilizing 19 shots over 1 hour. The target volume of the GK‐B was 976.8 mm^3^ and this received 100% of the prescription dose. The patient tolerated the procedure well and did not require steroids, pain medication or hospitalization. One week after completing GK‐SRS the patient started 33 fractions of IMRT. He received 60 Gy to the tumor bed and 57‐60 Gy to areas of subclinical disease risk. The positive margin disease below the GK‐B volume that included residual disease in the stylomastoid foramen received 66‐70 Gy. Treatment was delivered over 6.5 weeks with concurrent weekly cisplatin at 40 mg/m^2^.

**Figure 3:**
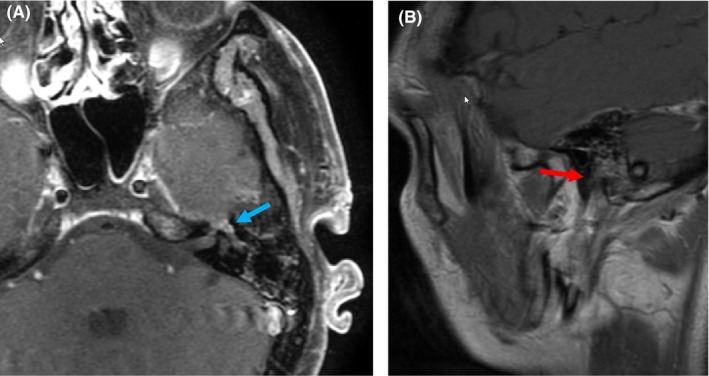
Follow‐up imaging status post completion of multimodality therapy. A, Axial and B, sagittal postcontrast, fat‐suppressed T1‐weighted MR images 12 months after completion of therapy, showing some mildly persistent but clearly diminished tumor enhancement in the left geniculate ganglion (blue arrow), and significantly regressed enhancement of the tympanic segment (compare with Figure 1C). There is near complete resolution of enhancing perineural tumor along the descending or mastoid segment (red arrow, compare with Figure 2B)

During treatment, the patient developed ipsilateral hearing loss on audiology testing. Review of the composite treatment plan revealed that the cochlea received a mean dose of 75 Gy. At 1‐year follow‐up, a MRI of the face and skull base revealed no evidence of locoregional recurrence (Figure 3A,B). The patient continued to have left‐sided hearing loss but no other treatment‐related toxicities.

## 3 DISCUSSION

This case demonstrates the effectiveness of GK‐B for ACC invading the skull base. The dosimetric advantage of GK‐SRS when compared to conventionally fractionated IMRT is its rapid dose fall off^3^ and has been shown to be safe in treating benign and malignant skull base tumors.^4^, ^5^ In addition, multiple studies have demonstrated low toxicity rates in patients with recurrent skull base disease reirradiated using SRS after prior external beam radiotherapy.^6^, ^7^ Patel et al demonstrated a 1‐year actuarial local control rate of 52.7% with a median OS of 25.4 months with the use of single‐fraction SRS for retreatment of recurrent skull base malignancies in a cohort of 18 patients.^8^ Only one patient in this study developed a significant radiation induced toxicity (late grade 2 radionecrosis).

**Figure 4:**
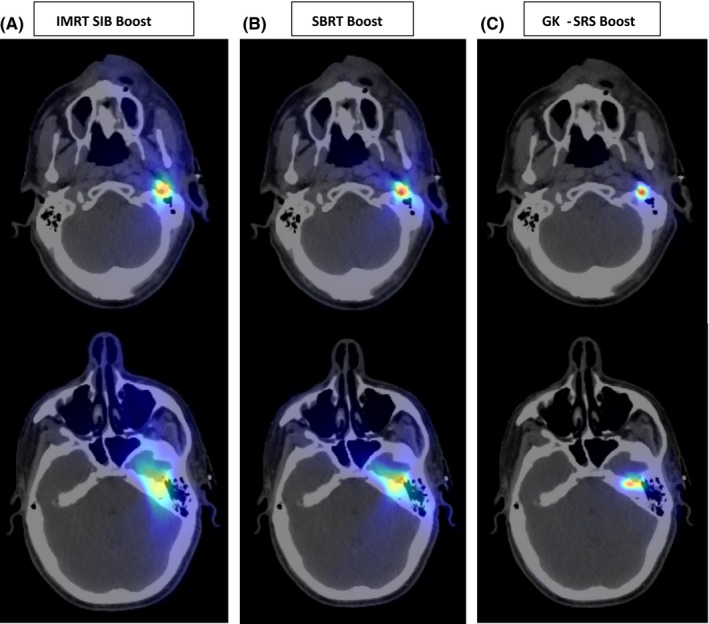
Dosimetric comparison of radiation treatment plans. A, Simulated IMRT 10 Gy boost with 3 mm PTV margin, B, SBRT 10 Gy boost with 1 mm PTV margin, and C, Simulated GK‐SRS 10 Gy boost targeting disease in stylomastoid foramen (top) and geniculate ganglion adjacent to temporal lobe (bottom). In comparison to other treatment pans, low dose cloud (blue) and intermediate dose clouds (yellow/green) are small. IMRT; intensity modulated radiation therapy, SBRT; stereotactic body radiation therapy, GK‐SRS; Gamma Knife stereotactic radiosurgery

During the treatment planning process for this patient, a plan comparison was performed to evaluate and determine the appropriate boost technique to address the gross disease in the skull base. We compared the composite plans of the conventionally fractionated IMRT plan (targeting the postoperative bed and residual skull base disease) combined with (a) a 10 Gy GK‐SRS boost, (b) a 10 Gy intensity modulated radiation therapy (IMRT) SIB boost (IMRT‐SIB), or (c) a 10 Gy linear accelerator based stereotactic body radiation therapy boost (SBRT‐B). The plans were evaluated with regards to maximum and mean dose to adjacent normal critical structures (Table 1). Both IMRT‐SIB and SBRT‐B plans were generated in Pinnacle (Pinnacle3, Phillips Medical Systems, Fitchburg, WI) using 6MV photon beams on a TrubeamTMSTx (Varian Medical Systems, Inc., Palo Alto, CA) linear accelerator with a two‐arc volumetric modulated arc therapy technique. As part of standard practice, a 3 mm planning target volume (PTV) margin was added for the IMRT‐SIB plan, a 1 mm margin was added for the SBRT‐B plan, and no margin was required for the GK‐B plan because stereotactic localization was utilized. In addition to a higher delivered dose to tumor, GK‐B demonstrates a more rapid dose fall off beyond the tumor and less volume of prescribed dose when compared to the IMRT‐SIB and SBRT‐B plans **(**Figure 4A‐C**)**. The use of GK‐SRS was chosen as the preferred boost modality for this patient.

**Table 1:** Dosimetric comparison between the three different composite plans

Organs at risk	GK‐SRS composite dose (Gy)	SBRT composite dose (Gy)	IMRT‐SIB composite dose (Gy)
Whole brain			
Maximum dose	73.8	75.3	76.1
Mean dose	5.1	5.1	5.1
Brainstem			
Maximum dose (point)	47.4	46.9	46.7
Dose (0.5 cc)	41.3	44.7	46.6
Mean dose	12.8	12.5	12.5
Left mandibular ramus			
Maximum dose	71.2	73.7	74.2
Mean dose	66.7	68.6	69.6
Left pharyngeal mucosa			
Maximum dose	70.1	72.9	73.1
Mean dose	50.9	52.2	52.4
Left temporal lobe			
Maximum dose (point)	76.0	78.0	76.5
Dose (1.0 cc)	66.4	72.1	76.3
Mean dose	22.6	22.3	23.2

IMRT, intensity modulated radiation therapy; GK‐SRS, Gamma Knife stereotactic radiosurgery; SBRT, stereotactic body radiation therapy.

As shown in Table 1, the GK**‐**B plan delivered less dose to normal structures than both the IMRT‐SIB and SBRT‐B plans, including lower maximum dose to the pharyngeal mucosa and mandibular ramus, as well as brain, brainstem, and temporal lobe. While it is universally agreed that lower doses to normal structures is preferable, it is unclear if the dose difference we observed in this comparison is clinically impactful. In the presented case, the patient tolerated therapy well and experienced limited acute and late complications after treatment.

To date, there are no prospective studies evaluating the dosimetric potential for toxicity reduction using GK‐SRS as a boost to address residual disease in the skull base for malignant tumors in comparison to the commonly utilized IMRT‐SIB approach. The authors hope this study will encourage further evaluation of GK‐SRS as boost therapy in cases of unresectable skull base disease. Ultimately, a larger sample size study and longer follow‐up are needed to evaluate the efficacy and long‐term toxicity profile of the GK‐SRS boost.

Here, we present a case of ACC of the left parotid gland with perineural spread through the left stylomastoid foramen to the ipsilateral geniculate ganglion. The patient was successfully treated with surgical resection followed by an upfront GK‐SRS boost to the skull base disease as a component of his overall adjuvant chemoradiation plan. We demonstrate favorable dosimetry using GK‐B with regards to the ipsilateral pharyngeal mucosa, mandibular ramus, temporal lobe, brainstem, and brain. At 1‐year follow‐up, the patient remains disease free and developed expected ipsilateral hearing loss based on the location of the tumor in proximity to the cochlea. This study illustrates the potential utility of GK‐SRS as a boost in high risk regions of the skull base to reduce unnecessary dose to critical structures such as the brainstem and temporal lobe.

## CONFLICTS OF INTEREST

None declared.

## AUTHORSHIP

Jae L. Phan: Primary author of paper and contributor to generation of figures, contributed to generation of concept for manuscript. Courtney Pollard: Major contributor to writing manuscript and generation of figures, contributed to generation of concept for manuscript. He Wang: Provided technical guidance for treatment descriptions in manuscript, helped design figures. Sweet Ping Ng: Contributed to generation of concept, secondary author, aided with revisions and editing. Tommy Sheu: Contributed to generation of concept, secondary author, aided with revisions and editing. Lawrence E. Ginsberg: Contributed to generation of concept, helped design figures, aided with revisions and editing. Amy Hessel: Contributed to generation of concept, aided with revisions and editing. Paul W. Gidley: Contributed to generation of concept, aided with revisions and editing. David I. Rosenthal: Contributed to generation of concept, aided with revisions and editing. Jack Phan: Generated concept for manuscript, major contributor to writing manuscript and generation of figures, primary editor and revisionist.
